# Midwifery students’ and teachers’ perceptions and needs regarding Extended Reality (XR) in educational training and its potential role in anxiety-related learning contexts

**DOI:** 10.18332/ejm/224204

**Published:** 2026-09-30

**Authors:** Clara Pérez de los Cobos Cintas, Nicolas Vuillerme, Lionel Di Marco, Guillaume Thomann

**Affiliations:** 1 Laboratoire des Sciences pour la Conception, l'Optimisation et la Production, Grenoble, France; 2 LIG Sangria, CNRS, Université Grenoble Alpes, Grenoble, France; 3 Institut Universitaire de France, Paris, France; 4 Department of Midwifery, Faculty of Medicine, Université Grenoble Alpes, Grenoble, France

**Keywords:** education, anxiety, training, midwifery, Extended Reality (XR)

## Abstract

**INTRODUCTION:**

Some midwifery students report anxiety or insufficient preparedness for clinical practice by the end of their initial education. Integrating Extended Reality (XR) into midwifery training may transform traditional teaching by offering immersive, interactive, and safe learning environments. While XR is becoming more common in healthcare education, a better understanding of how midwifery students and teachers perceive its utility, remains underexplored. This study aims to: 1) assess students’ and teachers’ expectations, needs, and experiences with the use of XR in training; and 2) explore perceptions of XR's potential to support training-related anxiety.

**METHODS:**

A cross-sectional survey was conducted among 86 students and 4 teachers from the Midwifery Department at Grenoble Alpes University, France. Participants rated the appropriateness of five XR training scenarios on a 10-point Likert scale, and later re-evaluated them considering XR’s potential for anxiety management. One open-ended question provided qualitative insights into expectations and perceived benefits.

**RESULTS:**

Participants viewed XR as an appropriate tool for midwifery education, emphasizing immersive, hands-on scenarios, such as gesture simulation and clinical case management. Emergency and crisis management emerged as the main use case (68%). When anxiety-related learning contexts were introduced as an additional consideration, participants’ evaluations remained consistent, suggesting that XR was perceived as relevant for high-pressure clinical training scenarios.

**CONCLUSIONS:**

Participants’ perceptions highlight the educational relevance of XR for simulation-based midwifery training, particularly in high-pressure clinical scenarios. These exploratory findings provide a basis for future studies involving direct XR exposure and validated outcome measures, and guided the development of an AR-based shoulder dystocia training module.

## INTRODUCTION

Midwifery education and training are intended to allow students to develop clinical decision-making skills through hands-on experience, as their profession requires independent work, a scientific approach, and a strong sense of professional responsibility^1^. However, previous research has shown that at the end of their initial education, nursing and midwifery students do not feel well prepared for clinical practice. They have expressed concerns about their competence, patient safety and risk of making mistakes during their first clinical placement, emphasizing the need for better preparation activities and support systems to increase their confidence and competence^2,3^.

Additionally, healthcare students are often exposed to stress and anxiety due to high workloads, significant responsibilities, and exposure to human suffering, among other factors^4^. To clarify the distinction between the two terms, often used interchangeably, stress is a process in response to situations, during which anxious reactions may occur. Stress refers to a psychological and physiological response to external pressures or demands, such as exams, work deadlines, or conflicts. It is a process that occurs when an individual perceives a situation as challenging or threatening^5^. Anxiety, on the other hand, is more closely related to the anticipation of perceived difficulty or threat, even when the threat is not immediate or specific. Anxiety often involves worrying or feeling uneasy about potential future events or outcomes^6^.

### Extended Reality (XR) state of the art

Interestingly, in recent years, several studies have focused on using Extended Reality (XR) in nursing and midwifery education to address these challenges^7^. In a recent systematic review, several studies suggested that XR training provides a more immersive and controlled learning environment, helping students manage stress and anxiety, as well as improving procedural confidence^8^.

XR is a term that includes all immersive technologies that merge the physical and digital settings, including Virtual Reality (VR), Augmented Reality (AR), and Mixed Reality (MR). VR includes environments where the user is fully immersed in the digital world. AR overlays digital information onto the real world, using devices such as smartphones, tablets, or AR headsets. Finally, by using MR, the user can interact with digital elements superimposed in the real world^9^.

Integrating XR technologies into midwifery education training has the potential to transform traditional teaching methods by offering immersive, interactive, and safe learning environments^10,11^. These tools enrich learning experiences, making them more engaging and effective while addressing specific educational needs. By using XR tools, students can simulate procedures and clinical decision-making without the fear of harming real patients, lowering their stress and improving procedural confidence^8,12,13^.

Another major advantage of XR technologies is their ability to expose students to new procedures and environments before they face them in reality. For instance, a 360-degree video of a Cesarean section allows students to be prepared for their first operating room intervention by familiarizing themselves with the procedure’s progression^14^ or with the room’s layout^15^. This type of immersive preparation helps students to build confidence and reduce anxiety. VR was used to teach anatomy courses^16^ and pathophysiology subjects^17^, helping learners understand complex concepts by providing three-dimensional visualization and interaction with the inside of the human body, which is not normally possible.

XR enables students to manage medical situations, such as recognizing errors in a virtual operating room^18^, and to practice communicating effectively with anxious patients in fully immersive virtual reality scenarios^19^. Another valuable application of XR technologies is the simulation and practice of essential gestures, such as those required for neonatal resuscitation^20^.

### Objectives and research question

It is important to better understand how future midwifery professionals perceive XR technology as they become increasingly common in healthcare education. To gain a deeper understanding of midwifery students’ needs, experiences, and expectations regarding the integration of XR technologies into their educational training, a questionnaire was specifically designed and administered.

The findings from this questionnaire form the basis for developing an XR tool, that is aligned with the specific needs and preferences of both students and teachers, providing relevance, usability, and educational effectiveness.

This work thus addresses the following two main research questions: ‘What are the expectations, needs and experiences of midwives’ students concerning the use of XR in their educational training?’ and ‘How do midwifery students and teachers perceive the potential of XR technologies in supporting learning in stressful or anxiety-provoking educational situations?’.

The findings will guide the development and evaluation of XR tools, combining technological innovation and the practical needs and concerns among midwives. Through appropriate XR integration, this work seeks to improve the teaching experience for future midwives by directly addressing expressed needs and innovative ideas for anxiety prevention.

## METHODS

Using a descriptive and mixed-methods approach that combines both quantitative and qualitative data, this study explored the perceptions of midwifery students regarding the use of XR technologies in educational training. A structured questionnaire was developed to collect data on participants’ perceptions of the appropriateness of XR in various learning scenarios. The survey aimed to explore participants’ perceptions of potential educational value of XR, including its anticipated usefulness in learning situations perceived as anxiety-related.

### Participants

The questionnaire was evaluated in the Midwifery Department of Grenoble Alpes University, France, in March 2024. Among 147 midwifery students across all levels of education and 6 teachers in the Department, 131 responses were registered. However, only 90 of these responses were complete and thus usable, resulting in a participation rate of 61.2%.

All participating students had prior experience with healthcare simulation training, including both low-fidelity and high-fidelity simulation as part of their midwifery curriculum, but had no previous exposure to XR technologies in either educational or clinical training contexts.

### Data collection process

The questionnaire had a 100% online format and participants were able to complete it from 7 to 19 March 2024.

### Survey design

At the beginning of the survey, participants were presented with the following project description:

‘A work opportunity with a laboratory developing Extended Reality (XR) tools makes it possible to consider deploying an XR learning tool for midwifery students. Extended reality is the umbrella term encompassing Augmented Reality (AR), Virtual Reality (VR), and Mixed Reality (MR) technologies. Augmented reality is the overlay of reality with elements (sounds, 2D or 3D images, videos, etc.) generated in real time by a computer system. In practical terms, it can enable the integration of virtual objects, text, or interactive features onto a screen displaying a live real-world environment. Virtual reality is a computer technology that simulates the physical presence of a user within an environment artificially generated by software. Virtual reality creates a virtual real or imaginary environment with which the user can interact.’

After the introduction, the questionnaire included five scenarios, each designed to measure participants’ perspectives on the appropriateness of using XR technologies in educational training. The respondents were asked to rate each scenario on a 10-point Likert scale (1=highly inappropriate to 10=highly appropriate). The scenarios were based on themes previously identified in the literature on the use of XR in nursing or midwifery studies and included: Discovery/visit of places^18,21^; Object manipulation/3D representation^22^; Management of human simulations^19^; Management of medical cases^23^; and Gesture realization/simulation^20,24,25^.

### Proposed scenarios

*Discovery/visit of places*

Exploring virtual environments or unknown locations through immersive XR experiences, for example visiting an operating room^18,21^.

*Object manipulation/3D representation*

Interacting with and manipulating virtual objects or 3D representations in an XR environment, such as anatomical structures^22^.

*Management of human simulations*

Utilizing XR for simulating and managing human interactions or scenarios, for example interacting with an anxious patient^19^.

*Management of medical cases*

Employing XR for training or simulating medical procedures and case studies, including scenarios such as patient safety training^23^.

*Gesture realization/simulation*

Simulating and practicing specific clinical gestures or movements within an XR environment, for example, performing neonatal resuscitation^20,24^ or suturing techniques^25^.

### Notion of anxiety

After rating these scenarios, participants were asked if they would change their responses if the scenarios were used to evaluate XR’s impact on student anxiety, focusing on working on particularly stressful situations. If so, they could re-evaluate the scenarios while considering this factor.

### Open-ended question

Finally, an open-ended question allowed respondents to provide suggestions or additional feedback regarding the use of XR in the midwifery educational context and anything that would be helpful in better understanding students’ and teachers’ needs. The question was as follows:

‘Thank you very much for taking part in this short survey. If you feel inspired, I invite you to describe in a few words in the box below what kind of project you think would be the most relevant to implement in this format. In other words, if you could make one (and only one) request that an immersive training module to be included in the midwifery curriculum, what would it be? You can mention the format it could take (immersion, computer-generated imagery, interactions …), the specific topic that should be addressed (e.g. handling a fetal mobile within the pelvis, team management during postpartum hemorrhage …), how it might be delivered technically (smartphone, VR headset …), and anything else you think would help clarify student needs.’

### Questionnaire validation

As no validated questionnaire was identified in the literature to assess users’ perceptions and needs regarding the use of XR in midwifery educational training, a dedicated questionnaire was specifically developed for this study. The questionnaire underwent content validity assessment by an expert panel^26^. The panel consisted of six experts: three lecturers from the Department of Midwifery and the Director of the Midwifery Program, all of whom are specialists in medicine, midwifery research, and medical simulation, as well as two lecturers and researchers with expertise in human-centered design engineering applied to the medical sector. The panel reviewed the questionnaire items and confirmed their relevance and appropriateness with respect to the current state of the art in XR technologies, as a part of the design process, and aligned with existing clinical training practices.

### Ethics

The study was conducted in accordance with local regulations for non-interventional studies in France, and did not require review by an Ethics Committee^27^. All participants were informed about the study’s aims, the voluntary nature of their participation, and the guarantee of anonymity. Data collection was strictly anonymous, ensuring that no identifying information was recorded. Participation in the survey was deemed informed consent.

### Data analysis

To analyze the data, a structured mixed-methods approach was followed, combining descriptive and inferential statistics for the Likert-scale questions and qualitative thematic analysis for the open-ended responses. Responses to Likert-scale items were summarized using means and standard deviations to describe participants’ perceptions and expectations regarding the use of XR in educational training. The results were analyzed by group (ESF2 to ESF5, and teachers) and by section of questions. Data were processed using Python.

Open-ended responses were reviewed and grouped according to recurring ideas and concepts. Themes were identified through an iterative process of response examination and categorization. These emergent themes were subsequently examined in relation to existing literature on XR applications in healthcare education and compared with the quantitative findings to assess consistency across data sources.

## RESULTS

To preserve anonymity and encourage participation, baseline demographic data such as sex, age, and other information about the participants, was not recorded, only their level of studies. The respondents included 21 midwifery students from the second year (ESF2) (23.3%), 23 from the third year (ESF3) (25.6%), 24 from the fourth year (ESF4) (26.7%), and 18 from the fifth year (ESF5) (20%), and 4 teachers (4.4%).

### Likert-scale questionnaire

All proposed scenarios received average ratings ranging from 5.6 ± 3.01 to 8.3 ± 2.26 on a Likert scale from 0 to 10, indicating overall positive perceptions of XR applications in midwifery education. The overall mean ± SD rating across all participants and scenarios was 6.9 ± 1.21, indicating a favorable attitude toward integrating XR technologies into midwifery training programs.

According to the survey, the most appropriate conditions for this project were: Management of medical situations and Simulation/execution of gestures, with average scores of 8.13 ± 2.32 and 8.3 ± 2.26, respectively.

### Scenario-based analysis

An in-depth analysis of the five proposed XR scenarios reveals different patterns in participant perceptions, as indicated by the mean scores and standard deviations (SDs), and medians and interquartile ranges (IQRs), summarized in Table 1 and represented in Figure 1. These statistical indicators not only reflect the overall evaluation of each scenario but also provide insight into the level of agreement or divergence among respondents.

**Table 1:** Ratings scores by scenario of exploratory cross-sectional survey results collected 7–19 March 2024 (N=90)

*Scenarios*	*Mean (SD)*	*Median (IQR)*
Discovery/visit of places	5.6 (3.01)	6.0 (3–9)
Object manipulation/3D representation	6.5 (2.32)	7.0 (5–8)
Management of human simulations	6.0 (2.72)	7.0 (4–8)
Management of medical cases	7.9 (2.11)	9.0 (7–10)
Gesture realization/simulation	8.3 (2.07)	9.0 (7–10)
All	6.9 (1.21)	8.0 (5–10)

**Figure 1:**
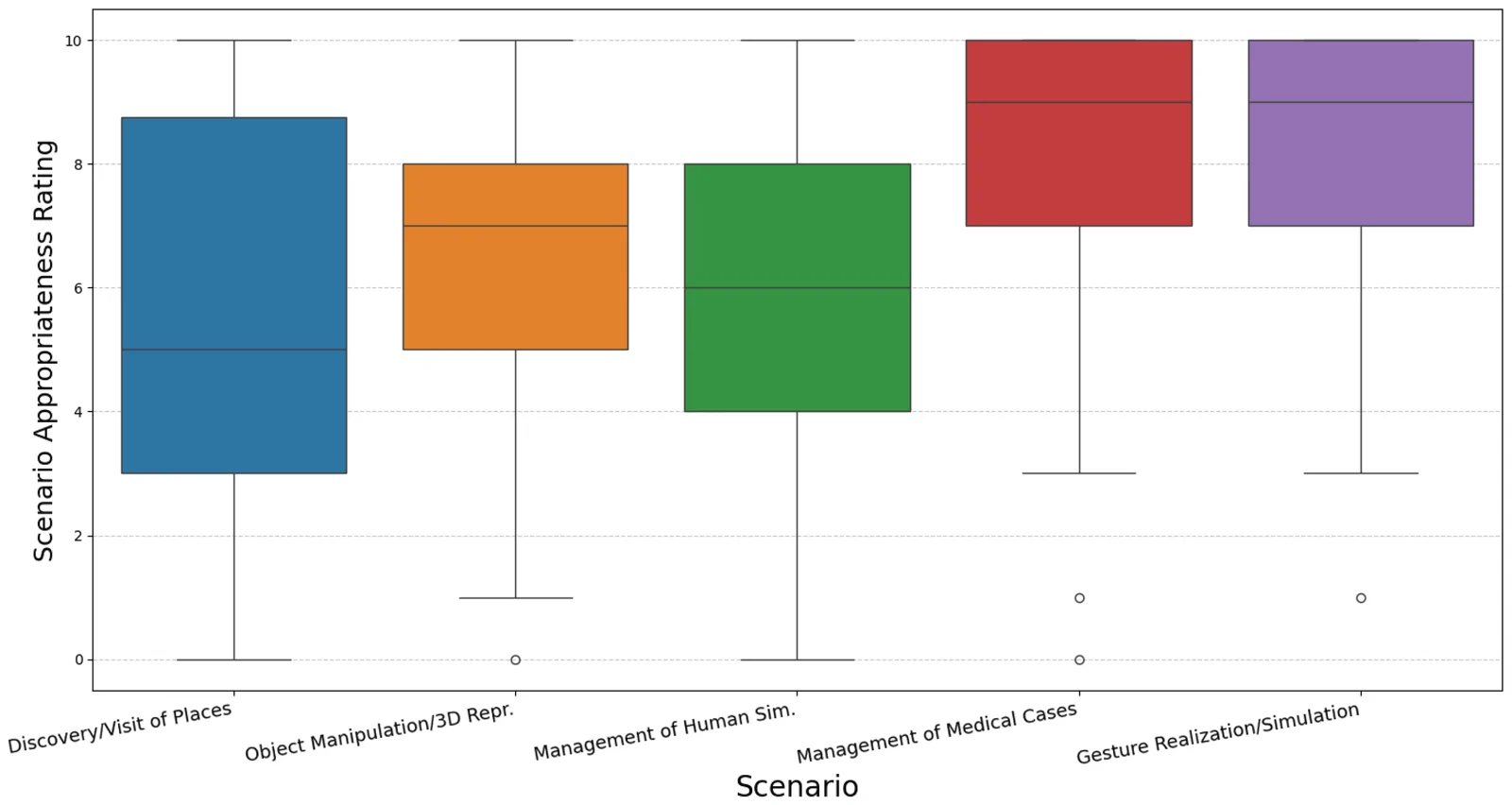
Appropriateness ratings scores by scenario of exploratory cross-sectional survey results collected 7–19 March 2024 (N=90)

The scenario Simulation/execution of gestures was the most highly rated and consistently valued application. It achieved a mean score of 8.3, a median of 9.0, a low standard deviation (SD=2.07), and a narrow interquartile range (IQR: 8–10), indicating strong agreement and high perceived relevance. Nearly half of the participants (48%) rated this scenario a 10 on the Likert scale, emphasizing its important role in supporting practical skill development, an essential component in midwifery training.

The scenario Management of medical cases also received notably high evaluations (mean=7.9, SD=2.11; median=9.0, IQR: 7–10), with 41% of participants assigning it the maximum score. The overall distribution suggests a broad recognition of the value of XR in enhancing decision-making and clinical reasoning through simulated casework.

In contrast, the scenario Object manipulation/3D representation received moderate but consistent evaluations (mean=6.5, SD=2.32; median=7.0, IQR: 5–8). This suggests that while not a top priority, such representations are still seen as useful tools for visualizing anatomical structures and complex procedures.

The scenario Management of human simulations showed greater variability in responses (mean=6.0, SD=2.72; median=7.0, IQR: 4–8) indicating more divided opinions. This could reflect different levels of familiarity with or expectations of human interaction simulations in a learning context. Additionally, some participants may perceive virtual human interaction simulations as less authentic, which could contribute to more divided opinions^28^.

Finally, the scenario Discovery/visit of places was the least well-rated (mean=5.6, SD=3.01; median=6.0, IQR: 3–9) with the highest variability across responses. This finding indicates that while some participants saw potential in virtual exploration, others questioned its relevance to midwifery skills.

### Group-based analysis

A descriptive subgroup analysis of the questionnaire responses was conducted to explore patterns in perceptions of the most appropriate use cases for XR in midwifery education. As 95% of the respondents were students and only 5% were teachers, comparisons between these groups should be interpreted with caution. The results are summarized in Table 2.

**Table 2:** Breakdown of scenario ratings scores (mean ± SD), by student group and teachers, of exploratory cross-sectional survey results collected 7–19 March 2024 (N=90)

*Scenarios*	*ESF2* *(N=21)*	*ESF3* *(N=23)*	*ESF4* *(N=24)*	*ESF5* *(N=18)*	*Teachers* *(N=4)*	*Students* *(N=86)*	*Total* *(N=90)*
Discovery/visit of places	6.57 ± 2.87	5.96 ± 3.29	4.5 ± 2.30	4.5 ± 3.20	8.0 ± 2.73	5.4 ± 3.17	5.6 ± 3.01
Object manipulation/3D representation	7.52 ± 2.63	6.87 ± 1.96	5.7 ± 2.42	5.83 ± 2.52	7.2 ± 2.16	6.5 ± 2.47	6.5 ± 2.32
Management of human simulations	6.09 ± 3.04	5.73 ± 3.4	7.42 ± 2.21	5.56 ± 3.09	4.4 ± 0.89	6.1 ± 2.96	6.0 ± 2.72
Management of medical cases	8.19 ± 2.08	7.82 ± 2.62	9.04 ± 1.16	7.67 ± 2.52	5.0 ± 1.22	8.2 ± 2.17	7.9 ±2.11
Gesture realization/simulation	8.95 ± 1.24	7.82 ± 2.44	9.21 ± 1.93	7.67 ± 2.49	5.2 ± 1.09	8.5 ± 2.15	8.3 ± 2.07
All	7.46 ± 1.16	6.84 ± 0.99	7.09 ± 2.06	6.24 ± 1.39	5.96 ± 1.51	6.94 ± 1.33	6.88 ± 1.21

On average, the scenario Discovery/visit of places received a score of 5.4 from students and 8.0 from teachers, resulting in an overall average of 5.6. The second scenario, Object manipulation/3D representation was rated 6.5 by the students, and 7.2 by the teachers, with an overall average of 6.5. For the scenario Management of human simulations, students gave an average score of 6.1, whereas teachers rated it lower at 4.4, leading to a combined average of 6.0. The scenario Management of medical cases was rated 8.1 by students and 5.0 by teachers, with a total average of 7.9. Finally, the scenario Gesture realization and simulation received the highest student rating, with an average of 8.5 compared with 5.2 from teachers, and a total average of 8.3.

The results vary depending on the respondent, as seen in Figure 2. Students showed a preference for experiential and action-based scenarios, particularly those simulating clinical complexity and tactile engagement. The top choices were Medical case management (averaging 8.22 ± 2.17) and Gesture simulation/execution (averaging 8.45 ± 2.15), which were identified by most students as the most appropriate applications of XR in educational training. While there was a slight variation depending on the course and experience of the students (years ESF2, ESF3, ESF4, and ESF5 of the Midwifery Department), the most voted results remained consistent.

**Figure 2:**
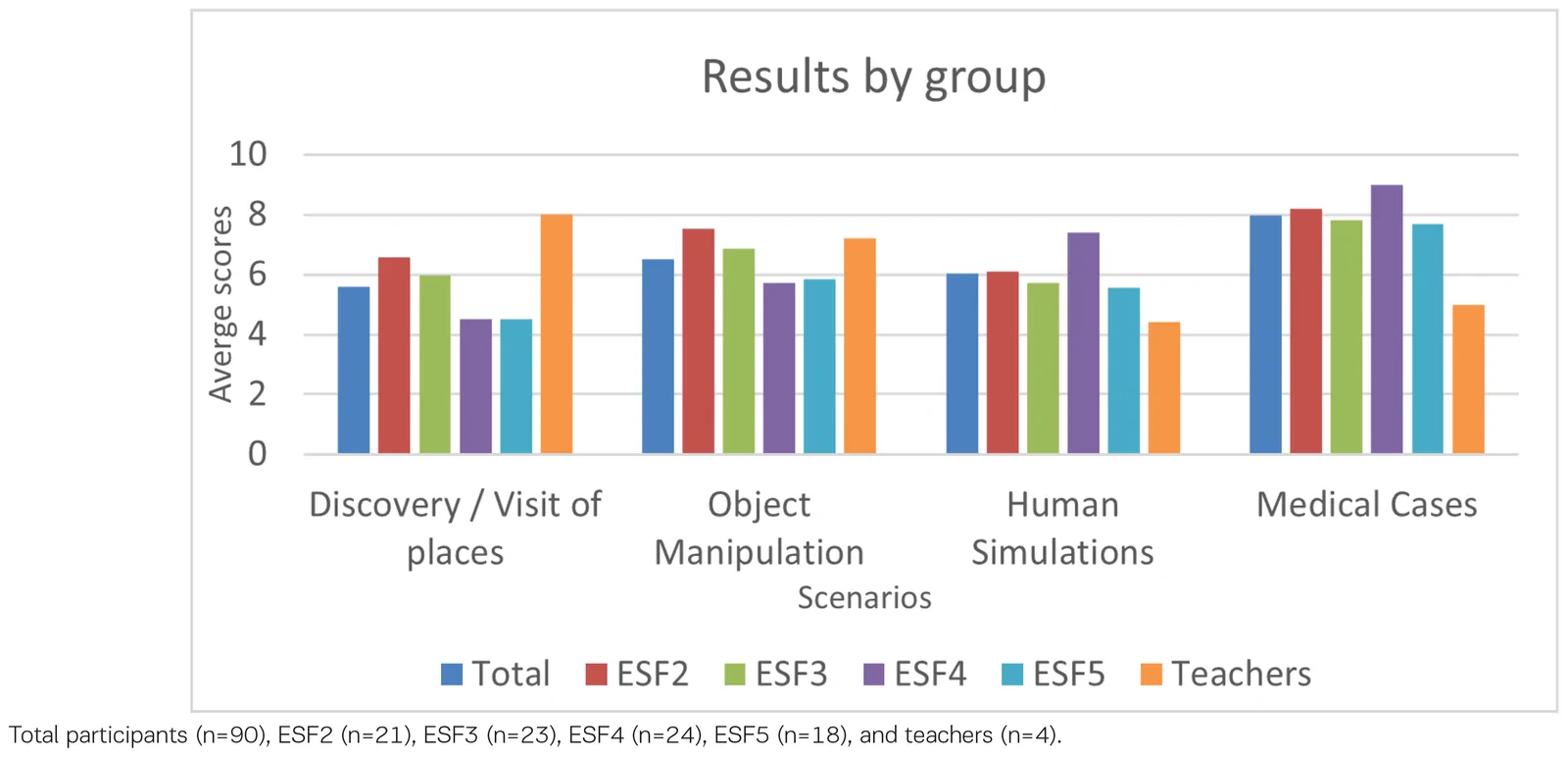
Average ratings scores of scenarios, by participant group, of exploratory cross-sectional survey results collected 7–19 March 2024 (N=90)

Within the teacher subgroup Discovery/visit of places and Manipulation of objects/3D representations, with average scores of 8 ± 2.73 and 7.2 ± 2.16, received the highest ratings. Overall, the mean of all the questions by group showed limited variation. The highest value was in ESF2 (7.46 ± 1.16), and the lowest was in ESF5 (6.94 ± 1.33), with an approximately half point difference.

### Introduction of the notion and definition of anxiety

Introducing the notion of anxiety had a minimal impact on participants’ perceptions regarding the most appropriate scenarios for the topic. Two respondents answered the question affirmatively, but they did not change their answers. Nine respondents (10%) changed their perspective after adding the notion of anxiety, 2 students from ESF2, 4 from ESF3, 3 from ESF5, and no teachers. The changed answers seemed to have slightly favor the situation [management of medical cases].

The within subject perspective shift (as shown in Table 3) allows us to evaluate the introduction of the notion of anxiety more in depth:

**Table 3:** Changes in the Likert-scale scenario mean ratings scores after the notion of anxiety was introduced for 9 students, of exploratory cross-sectional survey results collected 7–19 March 2024 (N=90)

*Scenarios*	*ESF2*	*ESF2*	*ESF3*	*ESF3*	*ESF3*	*ESF3*	*ESF5*	*ESF5*	*ESF5*	*Mean of changed responses*
Discovery/visit of places	5 to 8	8 to 8	3 to 7	9 to 8	9 to 3	10 to 10	2 to 7	6 to 10	3 to 7	7.5 ± 2.06
Object manipulation/3D representation	10 to 9	8 to 8	8 to7	9 to 9	6 to 6	9 to 9	2 to 4	5 to 7	3 to 6	7.2 ± 1.71
Management of human simulations	7 to 9	8 to 8	1 to 7	7 to 8	2 to 4	6 to 7	10 to 10	5 to 7	4 to 6	7.3 ± 1.73
Management of medical cases	7 to 10	8 to 8	3 to 10	8 to 9	6 to 9	8 to 9	10 to 10	10 to 10	4 to 6	9 ± 1.32
Gesture realization/simulation	7 to 10	8 to 8	8 to 8	10 to 10	5 to 3	5 to7	10 to 10	10 to 10	5 to 7	8.1 ± 2.31
Mean by group	7.6 ± 1.26 to 8.6 ± 0.84		6.4 ± 2.83 to 7.5 ± 2.14				5.93 ± 3.17 to 7.8 ± 2.01			6.84 ± 0.73

- Discovery/visit of places: 6/9 respondents increased their score, 1 stayed the same, 2 decreased.
- Object manipulation/3D representation: 4/9 increased, 4 stayed the same, and 1 decreased.
- Management of human simulations: all respondents increased or maintained their score.
- Management of medical cases: all 9 respondents either increased or maintained and strong increases (e.g. 3 to 10; 6 to 9) were present.
- Gesture realization/simulation: 5 increased, 3 maintained, and 1 decreased.

Overall, there was an increase in the average of all changed responses, indicating that respondents viewed the use of XR in teaching as more appropriate when it addressed anxiety-related situations.

### Open ended question

Twenty-nine participants (32%), including 3 teachers and 26 students (4 ESF2, 8 ESF3, 7 ESF4 and 7 ESF5), answered the open-ended question about what would be the most relevant projects to implement using XR tools.

A thematic analysis of the suggestions provided highlights a strong interest in integrating these tools, particularly Virtual Reality (VR), into midwifery and nursing education. The following themes emerged from the qualitative data, aligning with both the literature and the results of the Likert-scale questionnaire: emergency management, gesture realization/simulation, object manipulation/3D representation, team coordination, and the management of human simulations.

*Emergency situations and crisis management*

Emergency situations and crisis management was the theme most frequently mentioned by the respondents. Seventeen out of the twenty-nine respondents (68%) emphasized the value of immersive simulations to better prepare for rare yet critical scenarios, such as postpartum hemorrhage (PPH), shoulder dystocia, cardiac arrest, amniotic embolism, neonatal resuscitation, and red code emergency c-sections. These high-stakes situations, are often underrepresented in clinical training because of their unpredictability. XR tools can offer a more immersive learning experience, helping students feel better prepared to handle such emergencies. Previous studies have demonstrated the effectiveness of XR in this context, particularly for neonatal resuscitation^20,24^, or postpartum hemorrhage management^29^. Examples of responses from the students were:

*‘VR could simulate emergency situations such as hemorrhage, code red, cardiac arrest, and amniotic embolism so that we can be more comfortable handling them in real life.’* (ESF4)

*‘I would have liked to experience emergency scenarios during my training, especially those we rarely encounter as students, like postpartum hemorrhage or neonatal resuscitation.’* (ESF5)

*‘We need VR for technical maneuvers that are rarely done as students. Hemorrhage management, neonatal resuscitation, maternal resuscitation.’* (ESF3)

*‘Simulation of physiological delivery or rarely performed emergency procedures like postpartum hemorrhage, neonatal/maternal resuscitation. Ideally, with team-based simulation, VR offers more realistic spatial and role immersion than mannequins.’* (ESF3)

*‘VR lets us be fully immersed, including the physical stress, the gaze directions, and presence of others. All crucial in emergencies.’* (ESF4)

The teachers also stressed the importance of team-based emergency simulations:

‘VR should help with postpartum hemorrhage management by showing how *tools are organized around the patient.’* (Teacher)

*‘Simulation of postpartum hemorrhage management, absolutely essential.’* (Teacher)

*Gesture realization/simulation*

The theme of acquisition of technical skills includes student responses emphasizing the need for repeated, hand-son practice of clinical procedures. Previous studies have highlighted the value of XR for technical training, particularly for tasks such as suturing^25^. The students expressed a need to train for technical procedures that are difficult to practice and suggested the use of XR to increase both confidence and dexterity through immersive, repeatable training environments:

*‘Handling materials like IV pumps, Bakri balloons, sutures, urinary catheters. All of this could be practiced in XR to improve our speed and precision.’* (ESF4)

*‘Surgical sutures, internal monitoring like scalp pH or tocometry. They’re hard to explain and harder to simulate on mannequins.’* (ESF3)

*Object manipulation/3D representation*

Since XR technologies have been effectively used to teach disciplines such as anatomy^16,22^ or physiology^4,13^, this thematic grouping captures the way XR supports spatial and structural understanding of the human body. Students who identified as visual learners emphasized the benefit of 3D anatomical modeling to improve comprehension of pelvic anatomy, fetal positioning, and obstetric mechanics. Suggestions included:

*‘I need a 3D representation of the female reproductive system to visualize the cervix, pelvis shape, and fetal movement. It would help so much with my first vaginal exams and labor follow-ups.’* (ESF3)

*‘Using a VR headset to view obstetrical mechanics from every angle and position would show how important movement is in labor.’* (ESF5)

*‘It would be great to move around a semi-transparent pelvis and understand fetal movement better in 3D.’* (ESF5)

The students also mentioned the possibility of including 3D representation and emergency procedure understanding, which can also be found in the literature^10^:

*‘Mainly, I’d want to manipulate a fetal model in complicated cases, like breech birth or shoulder dystocia, using both hands tracked by controllers, like in VR games.’* (ESF3)

*‘Being able to visualize in 3D how the fetus moves through a semitransparent pelvis and practice Jaquemier or McRoberts maneuvers would be incredibly helpful.’* (ESF3)

*Coordination and delivery room workflow*

Similarly to what has been previously done in the literature related to coordination and room workflow^15^, one teacher provided a detailed vision of how VR could help simulate complex delivery ward scenarios, requiring users to coordinate multiple patients, staff, and emergencies in real-time. The simulation would incorporate multiple simultaneous labor and cesarean-section rooms, emergencies such as post-partum hemorrhage, pediatric and neonatal interventions and chronological pressure and task delegation. This type of scenario-based training could enhance decision-making under pressure, situational awareness, and interprofessional collaboration, skills essential for managing a dynamic clinical environment.

Another student suggested the possibility of using XR to learn how to work in a team of different specialists^30,31^ with teamwork training for operating room teams:

*‘It would be helpful to experience the organization of the team, what to expect before it happens during an internship, and how to react when it does.’* (ESF3)

*Management of human simulations*

The theme of management of human simulations reflects the potential of XR technologies to support the development of interpersonal competencies in high-pressure and emotionally complex clinical contexts^19^. Although some students expressed reservations about using VR to simulate human interactions, *‘Personally, I find it a shame to “simulate” human interaction situations’*. However, it would be interesting to be able to simulate stressful situations using a VR headset (ESF2). Others recognized its potential for training in stressful interpersonal situations since it could include managing emotionally complex consultations with patients or family members under pressure:

*‘Simulating difficult social situations with patients or couples (e.g. emotionally complex interactions).’* (ESF2)

## DISCUSION

The main trends identified in the Likert-scale questionnaire, the section addressing anxiety and the open question were aligned.

In the Likert-scale section, all the scenarios received average ratings ranging from 5.6 ± 3.01 to 8.3 ± 2.26 on a 1 (highly inappropriate) to 10 (highly appropriate) scale, indicating overall positive perceptions of XR applications in midwifery education. The highest rated scenarios were gesture simulation and medical case management, which appear to be perceived as the most relevant for training with these tools. Introducing the notion of anxiety did not significantly alter the ratings, suggesting that students’ and teachers’ views on the usefulness of XR were relatively stable regardless of this added dimension. However, in the open-ended question, the majority of the responses (68%) were related to emergency case management. This emphasis validates the expectations among teachers and students to simulate rare, unexpected and stressful situations. Such scenarios are often difficult to reproduce in traditional training, but are highly valued for preparing students to manage critical incidents, particularly with the immersive features that XR can provide.

The midwifery students and teachers who participated in our study expressed mostly consistent and complementary perceptions and opinions regarding the appropriateness of integrating XR technology into midwifery education. Their feedback provides valuable guidance for designing and implementing XR tools that align with educational needs.

Overall, the results of the present study point to the idea that scenarios focused on clinical practice, particularly those involving gesture simulation and medical case management, are perceived as the most useful, with high agreement. The lower and more variable ratings for exploratory or observational scenarios, such as the discovery of places or object manipulation, indicate uncertainty about their educational value or effectiveness when virtual elements are used. These findings support prioritizing immersive, clinically relevant XR experiences where interactivity is clearly recognized by learners as beneficial.

The results obtained suggest descriptive differences between the perceptions expressed by teachers and those reported by students, although these observations should be interpreted cautiously given the limited number of teacher participants (n=4). No significant differences were observed between student subgroups across academic years. Within this sample, teachers tend to place greater emphasis on structured learning experiences and conceptual understanding, prioritizing scenarios, such as discovery and visit of locations and manipulation of objects/3D representations. In contrast, students more frequently expressed interest in scenarios that imitate real-world clinical practice, including medical case management and gesture simulation/execution.

These descriptive trends may reflect the different educational priorities between teachers and students, and might be related to an important principle of active learning: teachers may not always have the relevant knowledge to anticipate what students will engage with actively (and therefore more effectively), and the effectiveness of implementing XR-based training depends largely on student engagement. While teachers’ opinions are important for ensuring pedagogical coherence and academic rigor, the effectiveness of XR-based training ultimately depends on whether students find the proposed scenarios motivating and immersive enough to adopt an active learning attitude. If learners do not perceive the activities as engaging or meaningful, their participation will remain passive, reducing the overall impact of the intervention^32^. The literature has already demonstrated how technology-enhanced learning has clear potential to enhance student engagement, promoting immersion and enjoyment^33^. Taken together, these findings suggest that the integration of XR into midwifery education may benefit from balancing foundational knowledge visualization with its potential for clinical skill acquisition and scenario-based learning, in an effort to narrow the gap between pedagogical intentions and learner engagement. The findings indicate that when anxiety is explicitly considered, more complex or emotionally intense scenarios are perceived as more appropriate for the use of XR, such as management of human simulations or medical cases. The scenario-by-scenario analysis is as follows.

*Discovery/visit of places*

When anxiety was considered, this scenario became more appropriate for most participants. XR might help reduce anxiety when unknown environments are explored virtually.

*Object manipulation/3D representation*

Participants found XR’s usefulness stable or slightly improved. Its direct link to anxiety management is less clear than that of more interpersonal or procedural scenarios.

*Management of human simulations*

Suggests that participants view XR simulations of human interactions as potentially helpful for managing stress/anxiety, possibly by allowing safer practice environments.

*Management of medical cases*

This scenario was highly valued when anxiety was considered. This likely reflects the high stress of real-life clinical decision-making, where XR could provide an opportunity for safe rehearsal.

*Gesture realization/simulation*

Practicing physical tasks (e.g. obstetric maneuvers) in XR is seen as more appropriate under anxiety. A single decrease (e.g. 5 to 3) could reflect a concern about realism or technical limitations.

Overall, the increase observed in the average of revised responses suggests that respondents perceived the use of XR in teaching as more appropriate when considering anxiety-related situations. However, the relatively low proportion of participants who modified their ratings (10%) indicates that the most of them had already formed stable opinions about the appropriateness of XR, even when anxiety was introduced as a factor. Several participants also commented that it would be relevant to include these technologies in courses on emergencies and complex clinical procedures, which are commonly perceived as stressful for the students. These findings reflect that while explicitly considering anxiety was not a major factor in changing participants’ opinions, they expressed the expectation that XR has the potential to support learning in complex and high-pressure training scenarios.

Thirty-two percent of the participants responded to the open-ended question, and among them, 68% identified emergency and crisis management as the most appropriate areas for XR application. This finding highlights a perceived need for using XR in training for complex, low-frequency or high-stakes scenarios.

Both students and teachers expressed support for XR tools in emergency simulation, technical skill development, anatomical visualization, and clinical decision-making. These qualitative insights, together with the Likert-scale questionnaire responses and based on the realities of their training and practice, reinforce the perceived value of integrating immersive technologies into the midwifery curriculum.

### Limitations

This study has several limitations that should be considered when interpreting the results. First, participants were recruited from a single institution, Grenoble Alpes University, France, which may limit the transferability of the results to other midwifery education settings or regions^34^. In addition, subgroup comparisons should be interpreted as exploratory, particularly given the limited number of teacher responses. The fully anonymous study design also restricted the collection of demographic data, limiting detailed characterization of the sample and a more extensive subgroup analysis. Second, the questionnaire was developed as an exploratory needs-assessment tool and has not undergone formal psychometric validation. Further validation is therefore required before its use in other educational contexts. However, involving students in the development of pedagogical content has been shown to improve learning outcomes and motivation^32^. As such, conducting a locally focused study remains appropriate for the scope of this project, even if the results cannot be extrapolated to national or international contexts without further investigation. Third, the data rely solely on self-reported perceptions and expectations, which are susceptible to social desirability bias or subjective interpretation^35^. Students may respond on the basis of what they think is expected or ideal, not necessarily their true experiences or opinions. Additionally, participants might have had limited or no direct exposure to XR technologies, and as mentioned, no exposure to XR technologies in their educational training. As a result, their understanding of the potential capabilities and limitations of these tools may be theoretical or speculative. Their expectations could be formed by secondhand information, assumptions, or general perceptions, rather than practical experience or the informed use of XR in educational contexts.

Importantly, this study did not involve any direct assessment of anxiety, nor did it evaluate the implementation of XR-based educational interventions. Consequently, no conclusions can be drawn regarding the actual effectiveness of XR for reducing anxiety or improving performance in stressful learning situations. Finally, the introduction of the notion of anxiety, after the initial scenario ratings might have caused a retrospective bias^36^, leading respondents to make superficial adjustments, as they had already formed an opinion about the technology’s appropriateness.

Despite these limitations, this exploratory study provides preliminary institution-specific insights into perceptions and expectations regarding XR integration in midwifery education, which may help inform future multicenter studies involving validated instruments and direct evaluation of XR-based educational interventions.

### Future research

This questionnaire was conducted in preparation for a follow-up study at the Midwifery Department at Grenoble Alpes University. Its purpose is to implement XR for uncertainty management in complex clinical situations during the upcoming academic year. Participants’ responses helped identify educational contexts in which XR was perceived as most relevant, guiding the design of an appropriate and useful tool for integration into midwifery studies.

Based on the questionnaire findings, XR integration was prioritized for practical training courses, specifically those focused on simulating obstetrical gestures or managing emergency situations.

The proposed solution was XR in shoulder dystocia training, where the students must recognize and handle this birth emergency, while performing the necessary maneuvers. This course is especially relevant because shoulder dystocia is both high-risk and anxiety-inducing for students, due to the limited practice (only a 2-hour session during their studies), and its potential for severe complications (including death, for both the mother and baby, if not managed properly). These characteristics make it an appropriate scenario for evaluating whether XR-based simulation may enhance technical skill acquisition, decision-making, learner confidence, and preparedness for high-pressure clinical situations.

## CONCLUSIONS

The present findings suggest that XR was generally perceived as a relevant and potentially useful tool for midwifery educational training. The midwifery students and teachers involved in the study expressed complementary perspectives on XR integration, highlighting its perceived potential to improve clinical training. Participants rated immersive, hands-on scenarios, such as gesture simulation and medical case management, as most valuable applications, as well as emergency and crisis management. Interestingly, although anxiety did not significantly shift participants’ opinions regarding XR technologies, they perceived XR as a supportive tool for learning complex and high-pressure training scenarios. However, these findings reflect perceptions and expectations based on hypothetical scenarios rather than direct experience with XR implementation, and therefore cannot support conclusions regarding its actual effectiveness for anxiety management or clinical performance improvement.

These findings support the need for future empirical studies involving direct XR exposure and validated outcome measures to evaluate its educational impact in midwifery training. They also supported the development of an AR shoulder dystocia training module at Grenoble Alpes University, translating the participants’ identified educational needs into a practical midwifery training application.

## Data Availability

The data supporting this research are available from the authors on reasonable request.
